# Atezolizumab and bevacizumab in patients with advanced hepatocellular carcinoma with impaired liver function and prior systemic therapy: a real-world experience

**DOI:** 10.1177/17588359221080298

**Published:** 2022-02-26

**Authors:** Tiago de Castro, Leonie S. Jochheim, Melanie Bathon, Sabrina Welland, Bernhard Scheiner, Kateryna Shmanko, Daniel Roessler, Najib Ben Khaled, Matthias Jeschke, Johannes M. Ludwig, Jens U. Marquardt, Arndt Weinmann, Matthias Pinter, Christian M. Lange, Arndt Vogel, Anna Saborowski

**Affiliations:** Department of Gastroenterology, Hepatology and Endocrinology, Hannover Medical School, Hannover, Germany; Department of Gastroenterology and Hepatology, Essen University Hospital, Essen, Germany; Department of Gastroenterology, Hepatology and Endocrinology, Hannover Medical School, Hannover, Germany; Department of Gastroenterology, Hepatology and Endocrinology, Hannover Medical School, Hannover, Germany; Division of Gastroenterology & Hepatology, Department of Internal Medicine III, Medical University of Vienna, Vienna, Austria; Department of Internal Medicine I, University Medical Center of the Johannes Gutenberg University Mainz, Mainz, Germany; Department of Medicine II, University Hospital of the Ludwig-Maximilians-University Munich, Munich, Germany; Department of Medicine II, University Hospital of the Ludwig-Maximilians-University Munich, Munich, Germany; Department of Gastroenterology and Hepatology, Essen University Hospital, Essen, Germany; Institute of Diagnostic and Interventional Radiology and Neuroradiology, Faculty of Medicine, Essen University Hospital, Essen, Germany; Department of Medicine I, University Hospital Schleswig-Holstein, Lübeck, Germany; Department of Internal Medicine I, University Medical Center of the Johannes Gutenberg University Mainz, Mainz, Germany; Division of Gastroenterology & Hepatology, Department of Internal Medicine III, Medical University of Vienna, Vienna, Austria; Department of Gastroenterology and Hepatology, Essen University Hospital, Essen, Germany; Department of Gastroenterology, Hepatology and Endocrinology, Hannover Medical School, Carl-Neuberg-Straße 1, 30625 Hannover, Germany; Department of Gastroenterology, Hepatology and Endocrinology, Hannover Medical School, Hannover, Germany

**Keywords:** Albumin-bilirubin score, atezolizumab, bevacizumab, hepatocellular carcinoma, IMbrave150 trial, PD-L1 inhibitor

## Abstract

**Objective:**

Evaluation of the efficacy and safety of atezolizumab/bevacizumab in a real-world HCC cohort, including patients with impaired liver function and prior systemic therapy.

**Methods:**

Retrospective analysis of 147 HCC patients treated with atezolizumab/bevacizumab at six sites in Germany and Austria.

**Results:**

The overall response rate and disease control rate were 20.4% and 51.7%, respectively. Seventy-three patients (49.7%) met at least one major exclusion criterion of the IMbrave150 trial (IMbrave-OUT), whereas 74 patients (50.3%) were eligible (IMbrave-IN). Median overall survival (mOS) as well as median progression-free survival (mPFS) was significantly longer in IMbrave-IN *versus* IMbrave-OUT patients [mOS: 15.0 months (95% confidence interval (CI): 10.7–19.3] *versus* 6.0 months (95% CI: 3.2–8.9; *p* < 0.001) and mPFS: 8.7 months (95% CI: 5.9–11.5) *versus* 3.7 months (95% CI: 2.7–4.7; *p* < 0.001)]. Prior systemic treatment did not significantly affect mOS [hazard ratio (HR): 1.32 (95% CI: 0.78–2.23; *p* = 0.305)]. mOS according to ALBI grades 1/2/3 were 15.0 months (95% CI: not estimable), 8.6 months (95% CI: 5.4–11.7), and 3.2 months (95% CI: 0.3–6.1), respectively. ALBI grade and ECOG score were identified as independent prognostic factors [ALBI grade 2 *versus* 1; HR: 2.40 (95% CI: 1.34 – 4.30; *p* = 0.003), ALBI grade 3 *versus* 1; HR: 7.28 (95% CI: 3.30–16.08; *p* < 0.001), and ECOG ⩾2 *versus* 0; HR: 2.09 (95% CI: 1.03 – 4.23; *p* = 0.042)], respectively. Sixty-seven patients (45.6%) experienced an adverse event classified as CTCAE grade ⩾3. Patients in the IMbrave-OUT group were at increased risk of hepatic decompensation with encephalopathy (13.7% *versus* 1.4%, *p* = 0.004) and/or ascites (39.7% *versus* 9.5%; *p* < 0.001).

**Conclusion:**

In this real-world cohort, efficacy was comparable to the results of the IMbrave150 study and not affected by prior systemic treatment. ALBI grade and ECOG score were independently associated with survival. IMbrave-OUT patients were more likely to experience hepatic decompensation.

## Introduction

Hepatocellular carcinoma (HCC) is the fifth most common cancer worldwide and was the third leading cause of cancer-related death in 2020.^1^ HCCs frequently develop in patients with pre-existing chronic liver diseases, and an impaired liver function often influences and complicates treatment decisions.^2^ Although curative treatment options for early-stage HCC such as surgical resection, transplantation, and ablation are available, up to 70% of patients experience tumor recurrence within 5 years.^3^ Despite the available screening opportunities for patients at risk,^4,5^ most patients are ineligible for curative therapies at diagnosis and eventually require systemic therapy.^6^ Following its approval in 2007, the multikinase inhibitor sorafenib remained the only treatment for patients with non-resectable HCC over almost a decade.^7^ In recent years, the approval of additional systemic treatment options resulted in a restructuring of HCC therapy concepts.^8,9^ Based on the positive results from the IMbrave150 trial,^10^ the combination of the programmed death ligand-1 (PD-L1) inhibitor atezolizumab and the vascular endothelial growth factor (VEGF) inhibitor bevacizumab is now considered the new standard of care for first-line treatment of patients with advanced HCC.^9,11^

Due to the inherently worse prognosis of patients with advanced cirrhosis, patients with Child–Pugh score (CPS) >A are generally excluded from pivotal trials, including IMbrave150, to avoid confounding results.^12^ Importantly, baseline liver function is not only a prognostic factor for overall survival (OS) in patients with HCC, but might also impact the efficacy and safety of systemic treatments.^13^ The clinical benefit and the safety profile of atezolizumab/bevacizumab have not yet been fully assessed in patients with impaired liver function and/or advanced treatment lines.

In retrospective and prospective cohorts, an overall survival of only 2.5–5.2 months was reported for CPS B patients under sorafenib,^14–19^ albeit safety and tolerability were comparable to CPS A patients.^14,16^ Regarding immune-checkpoint inhibitors (ICI), nivolumab has been tested in patients with impaired liver function (CPS B7-B8) in the prospective phase I/II CheckMate 040 trial,^20^ with an encouraging median overall survival (mOS) of 9.8 and 7.3 months in sorafenib-naive (*n* = 25) and sorafenib-experienced (*n* = 24) patients, respectively. The investigator-assessed overall response rate (ORR) was 12% [95% confidence interval (CI): 5–25%] and the disease control rate (DCR) reached 55% (95% CI: 40–69%) with no major differences in terms of safety in CPS A *versus* B patients. Survival of patients treated with ICI in real-world cohorts appears to be similar (mOS up to 9.6 months), and no major safety concerns have been reported.^21–24^

In addition to CPS, the albumin-bilirubin (ALBI) score, which is calculated using only serum albumin and total bilirubin, has been developed as an objective tool to assess liver function in HCC patients.^25^ Of note, ALBI score facilitates a more granular discrimination of the CPS A population, which resulted in the implementation of the ALBI score as a stratification factor in most ongoing clinical trials.

The aim of this study was to investigate the efficacy and safety of atezolizumab/bevacizumab for advanced HCC in a real-world cohort, including patients with impaired liver function, impaired performance status, and after prior systemic therapies.

## Patients and methods

### Patient selection

This study was designed as a retrospective case series of patients with advanced HCC who received atezolizumab 1200 mg plus bevacizumab 15 mg/kg body weight intravenously every 3 weeks in six tertiary academic hospitals in Germany (Hannover Medical School, Essen University Hospital, University Medical Center of the Johannes Gutenberg University Mainz, University Hospital of the Ludwig-Maximilians-University Munich, Campus Lübeck of the University Medical Center Schleswig-Holstein) and Austria (Medical University of Vienna) (Supplemental Table S1).

Treatment decisions were based on the recommendations of the local interdisciplinary tumor boards, and patients were deemed ineligible for surgical resection, locoregional therapy, or liver transplantation. Inclusion in the analysis was independent of previous systemic therapies. In eight patients, bevacizumab was withheld until upper endoscopy was performed. Liver function was assessed according to CPS and ALBI grade. Patients were classified according to Barcelona Clinic Liver Cancer (BCLC) system and were further grouped into two sub-cohorts: those who met the inclusion criteria of the IMbrave150 trial^10^ [CPS A, Eastern Cooperative Oncology Group (ECOG) performance status 0 or 1, and were therapy-naive for systemic HCC-specific treatment = IMbrave-IN group] and those who met at least one major exclusion criterion of the IMbrave150 trial (patients with a CPS ⩾B7, ECOG ⩾2, or who had received prior systemic therapies = IMbrave-OUT group).

Baseline characteristics, including age, sex, weight, underlying liver disease, and tumor-specific characteristics such as BCLC stage, macrovascular invasion, extrahepatic spread, and previous treatments, were collected retrospectively. Adverse events (AE) were graded according to the common terminology criteria for adverse events (CTCAE) Version 5.0.^26^ Changes in liver function were assessed by comparing CPS and ALBI score at baseline and thereafter every 8–12 weeks until the end of treatment. Treatment with atezolizumab/bevacizumab was continued until tumor progression or intolerance, including worsening of liver function (i.e. worsening of ascites and/or hepatic encephalopathy requiring hospitalization).

Tumor responses were assessed by computed tomography or magnetic resonance imaging at baseline and thereafter every 8–12 weeks until treatment was stopped. The best radiological response was classified as complete or partial response, stable disease, or progressive disease by local review.

### Statistical analysis

All statistical analyses were performed using IBM SPSS Statistics for Macintosh, Version 28.0 (IBM Corp. Released 2021, Armonk, NY). *P*-values <0.05 were considered statistically significant. Data were expressed as number/percentage, mean, or median. Differences between categorical variables were calculated using Pearson’s Chi-square or Fisher’s exact test, whenever appropriate. Changes in ALBI scores were analyzed using the Wilcoxon signed-rank test. Both mOS and median progression-free survival (mPFS) were computed using Kaplan–Meier curves and compared with the Mantel-cox log-rank test. Hazard ratios for events were estimated using univariable and multivariable Cox’s regression analysis using a stepwise backward elimination, with exclusion of variables with *p*-value >0.10. Only patients who received at least one dose were included in safety analysis. Patients were followed until death or date of data cut-off (19 November 2021).

## Results

Between November 2019 and November 2021, a total of 155 patients with advanced HCC were treated with atezolizumab/bevacizumab with the exception of one patient who received only atezolizumab due to severe hemophilia A with a high risk of bleeding. Eight patients were excluded from further analyses due to incomplete liver function data that prevented adequate assessment of baseline CPS and/or ALBI score. In all, 147 patients were included in the final analysis and classified as IMbrave-IN (*n* = 74) or IMbrave-OUT (*n* = 73). Clinical parameters at baseline are summarized in Table 1.

**Table 1.** Baseline characteristics according to patients’ eligibility for the IMbrave150 trial.

	IMbrave-IN (*n* = 74) *n* (%)	IMbrave-OUT (*n* = 73) *n* (%)	Total (*n* = 147) *n* (%)	
Age (years)				
Mean (range)	69.1 (38–88)	67.2 (30–96)	68.7 (30–96)	
Sex				
Male	61 (82.4)	64 (87.7)	125 (85.0)	
ECOG score				
0	33 (44.6)	16 (21.9)	49 (33.3)	
1	41 (55.4)	33 (45.2)	74 (50.3)	
2	0 (0)	23 (31.5)	23 (15.6)	
3	0 (0)	1 (1.4)	1 (0.7)	
Cirrhosis				
Present	54 (73.0)	62 (84.9)	116 (78.9)	
BCLC stage				
BCLC A	1 (1.4)	0 (0.0)	1 (0.7)	
BCLC B	14 (18.9)	9 (12.3)	23 (15.6)	
BCLC C	59 (79.7)	57 (78.0)	116 (78.9)	
BCLC D	0 (0)	7 (9.6)	7 (4.8)	
Child–Pugh score				
Child–Pugh A	74 (100)	32 (43.8)	106 (72.1)	
Child–Pugh B	0 (0)	35 (47.9)	35 (23.8)	
Child–Pugh C	0 (0)	6 (8.2)	6 (4.1)	
ALBI grade				
ALBI 1	42 (56.8)	9 (12.3)	51 (34.7)	
ALBI 2	32 (43.2)	51 (69.9)	83 (56.5)	
ALBI 3	0 (0)	13 (17.8)	13 (8.8)	
Etiology				
ASH	16 (21.6)	23 (31.5)	39 (26.5)	
Hepatitis C	21 (28.4)	17 (23.3)	38 (25.9)	
Hepatitis B	2 (2.7)	10 (13.7)	12 (8.2)	
NASH	22 (29.7)	12 (16.4)	34 (23.1)	
Other	1 (1.4)	3 (4.1)	4 (2.7)	
Unknown	12 (16.2)	8 (11.0)	20 (13.6)	
Presence of macrovascular invasion / extrahepatic spread				
MVI	23 (31.5)	25 (34.7)	48 (33.1)	
EHS	37 (50.0)	28 (38.9)	65 (44.5)	
MVI, EHS, or both	60 (81.1)	53 (72.6)	113 (76.9)	
AFP				
AFP ⩾400 ng/ml	22 (30.6)	30 (41.1)	52 (35.9)	
Prior local therapy				
At least one prior local therapy	32 (43.2)	34 (46.6)	66 (44.9)	
Resection	12 (16.2)	13 (17.8)	25 (17.0)	
Ablation	7 (9.5)	10 (13.7)	17 (11.6)	
TACE	18 (24.3)	24 (32.9)	42 (28.6)	
SIRT	12 (16.2)	6 (8.2)	18 (12.2)	
Radiation	1 (1.4)	1 (1.4)	2 (1.4)	
Prior systemic therapy				
⩾1 line of systemic therapy	0 (0)	29 (39.7)	29 (19.7)	
1st line sorafenib	0 (0)	18 (24.7)	18 (12.2)	
1st line ICI	0 (0)	7 (9.6)	7 (4.8)	
1st line lenvatinib	0 (0)	3 (4.1)	3 (2.0)	
1st line combination^a^	0 (0)	1 (1.4)	1 (0.7)	
⩾2 lines of systemic therapy	0 (0)	17 (23.3)	17 (11.6)	
Esophageal varices at baseline				
Screened	61 (82.4)	51 (70.0)	112 (76.2)	
Absence of varices	34 (45.9)	19 (26.0)	53 (36.1)	
Grade I or II	23 (31.1)	29 (39.7)	52 (35.4)	
Grade II with red spots or III	4 (5.4)	3 (4.1)	7 (4.8)	
Not screened	13 (17.6)	22 (30.1)	35 (23.8)	
Received treatment for esophageal varices, of which:	23 (31.1)	23 (31.5)	46 (31.3)	
Oral non-selective beta-blocker	17 (23.0)	14 (19.2)	31 (21.1)	
Grade I or II	16 (21.6)	14 (19.2)	30 (20.4)	
Grade II with red spots or III	1 (1.4)	0 (0.0)	1 (0.7)	
Banding	5 (6.8)	9 (12.3)	14 (9.5)	
Grade I or II	2 (2.7)	6 (8.2)	8 (5.4)	
Grade II with red spots or III	3 (4.1)	3 (4.1)	6 (4.1)	

AFP, alpha-fetoprotein; ALBI, albumin-bilirubin; ASH, alcoholic steatohepatitis; BCLC, Barcelona Classification Liver Cancer; ECOG, Eastern Cooperative Oncology Group; EHS, extrahepatic spread; ICI, immune checkpoint inhibitor; MVI, macrovascular invasion; NASH, non-alcoholic steatohepatitis; no., number; SIRT, selective internal radiation therapy; TACE, transarterial chemoembolization

a Combination therapy included spartalizumab and sorafenib.

Most patients were male (85.0%) and the mean age at treatment start was 68.7 years (range, 30–96 years). 116 patients (78.9%) had been diagnosed with cirrhosis, with no significant differences between both subgroups. Liver function was assessed based on CPS and ALBI scores. 41 patients (27.9%) presented with an impaired liver function (defined as CPS ⩾B7), while stratification according to ALBI score revealed an advanced liver dysfunction (ALBI grade ⩾2) in more than half of the cohort (*n* = 96, 65.3%). The majority of CPS A5 patients were classified as ALBI grade 1, while the majority of CPS A6 patients were ALBI grade 2 (Table 2).

**Table 2.** Distribution of patients according to Child–Pugh score and ALBI grade.

		ALBI 1 *n* (%)	ALBI 2 *n* (%)	ALBI 3 *n* (%)
CPS A	A5	46 (90.2)	26 (31.3)	0 (0)
	A6	4 (7.8)	30 (36.1)	0 (0)
CPS B	B7	1 (2.0)	16 (19.3)	1 (7.7)
	B8	0 (0)	9 (10.8)	3 (23.1)
	B9	0 (0)	2 (2.4)	3 (23.1)
CPS C	C10	0 (0)	0 (0)	4 (30.8)
	C11	0 (0)	0 (0)	2 (15.4)

ALBI, albumin-bilirubin; CPS, Child–Pugh score.

Baseline characteristics of both subgroups did not differ significantly with respect to macrovascular invasion, extrahepatic spread, disease stage B and C according to BCLC, and etiology. One 88-year-old patient was deemed unfit for surgical resection or local therapy and received atezolizumab/bevacizumab despite a BCLC A situation. Six CPS C patients and one patient classified as ECOG 3 were treated with combination therapy based on individual decision by the treating physician; while the CPS C patients were of comparatively young age (mean, 64 ± 4.2 years) and mainly ECOG 1, the ECOG 3 patient had a preserved liver function CPS A5 and no cirrhosis. The number of patients with increased baseline alpha-fetoprotein (AFP) levels ⩾400 ng/ml was similar in the IMbrave-IN and IMbrave-OUT subgroups.

Patients in both subgroups had undergone locoregional anticancer procedures at a similar rate (43.2% *versus* 46.6% in the IMbrave-IN and the IMbrave-OUT subgroup, respectively). Twenty-nine patients (39.7%) in the IMbrave-OUT subgroup had received prior systemic therapies. Sorafenib was the most common drug used in first-line (*n* = 18, 24.7%), followed by ICI in 7 (9.6%) patients (nivolumab *n* = 6; tislelizumab *n* = 1) and lenvatinib (*n* = 3, 4.1%). One patient was treated with spartalizumab in combination with sorafenib within a clinical trial (Table 1).

A total of 112 patients (76.2%) had undergone screening for esophageal varices by upper endoscopy within 6 months prior to treatment initiation, of which 59 patients (40.1%) were diagnosed with esophageal varices: 52 patients (35.4%) with varices grade I or II without red spots (low-risk group) and 7 patients (4.8%) with varices grade II with red spots or grade III (high-risk group). Oral non-selective beta-blockers had been started in 30 patients (20.4%) of the low-risk group and in 1 patient (0.7%) with high-risk varices, and banding therapy had been performed in 8/52 patients with low-risk varices and in 6/7 patients (4.1%) with high-risk varices. IMbrave-OUT patients were more likely to present with esophageal varices of any grade (62.7% *versus* 44.3%, *p* = 0.05). However, there was no major difference in terms of severity of baseline varices, type of varices treatment and probability for varices screening in the IMbrave-IN *versus* IMbrave-OUT group (Table 1).

## Efficacy analysis

Patient disposition is summarized in Table 3. The median follow-up was 6.2 months (range, 0.4–22.2 months) and the median number of administered atezolizumab/bevacizumab treatments was 6 (range, 1–27). At the time of last follow-up, more patients in the IMbrave-OUT subgroup had discontinued treatment (82.2% *versus* 68.9%; *p* = 0.06) and a significantly higher rate of treatment discontinuation due to liver function deterioration was evident (13.7% *versus* 2.7%; *p* = 0.02) compared with IMbrave-IN patients.

**Table 3.** Patient disposition according to patients’ eligibility for the IMbrave150 trial.

Patient disposition	IMbrave-IN (*n* = 74) *n* (%)	IMbrave-OUT (*n* = 73) *n* (%)	Total (*n* = 147) *n* (%)
Median follow-up time in months (range)	7.3 (0.8–22.2)	4.4 (0.4–19.3)	6.2 (0.4–22.2)
Median no. of cycles of atezolizumab/bevacizumab (range)	8 (1–27)	5 (1–22)	6 (1–27)
Treatment ongoing	23 (31.1)	13 (17.8)	36 (24.5)
End of treatment	51 (68.9)	60 (82.2)	111 (75.5)
Progression	33 (44.6)	37 (50.7)	70 (47.6)
Adverse events	10 (13.5)	8 (11.0)	18 (12.2)
Liver function deterioration	2 (2.7)	10 (13.7)	12 (8.2)
Patient decision	0 (0.0)	2 (2.7)	2 (1.4)
Lost to follow-up	2 (2.7)	0 (0.0)	2 (1.4)
Death (undetermined cause)	1 (1.4)	3 (4.1)	4 (2.7)
Regular end after complete response	3 (4.1)	0 (0.0)	3 (2.0)

**Figure 1.**
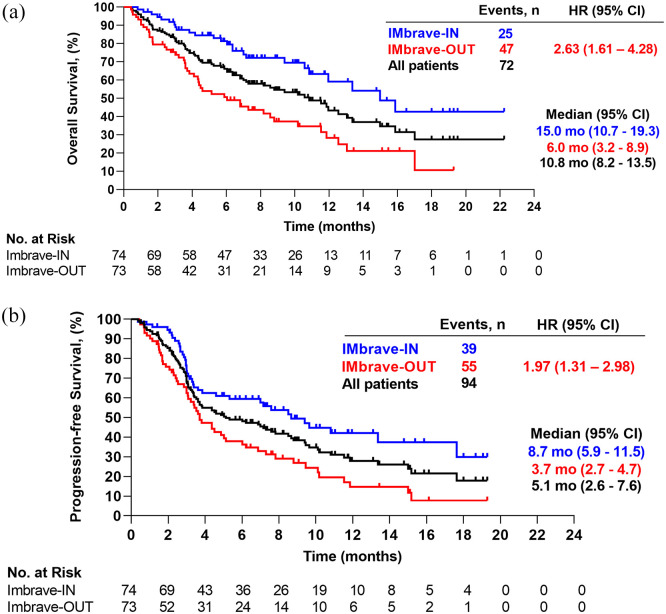
Kaplan–Meier analysis of (a) overall survival and (b) progression-free survival of patients treated with atezolizumab/bevacizumab according to IMbrave criteria. IMbrave-IN: patients who would have met the inclusion criteria of the IMbrave150 trial. IMbrave-OUT: patients who met at least one major exclusion criterion of the IMbrave150 trial. CI, confidence interval; HR, hazard ratio; mo, months; No., number.

**Figure 2.**
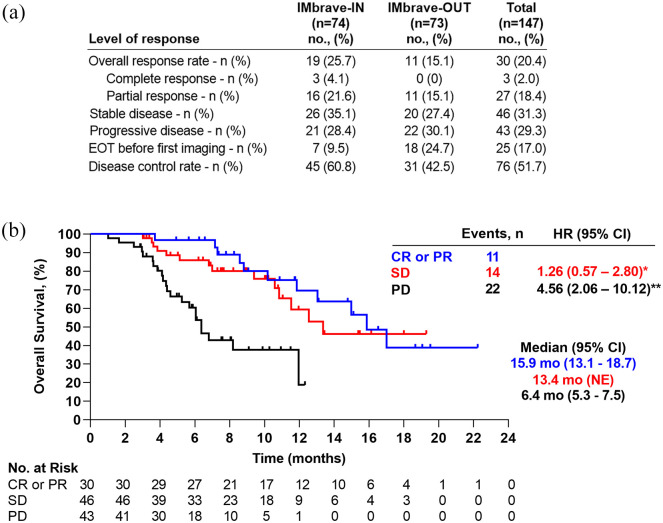
(a) Patient distribution according to response assessment in the IMbrave-IN and IMbrave-OUT cohorts. (b) Kaplan–Meier analysis of overall survival according to radiologic response under treatment with atezolizumab/bevacizumab. CI, confidence interval; CR, complete response; EOT, end of treatment; HR, hazard ratio; mo, months; NE, not estimable; No., number; PD, progressive disease; PR, partial response; SD, stable disease. *Comparing HR of death between patients with CR or PR and SD; **comparing HR of death between patients with CR or PR and PD.

The mOS for the whole cohort was 10.8 months (95% CI: 8.2–13.5) and the mPFS was 5.1 months (95% CI: 2.6–7.6) (Figure 1(a) and (b)). Patients in the IMbrave-IN subgroup had a significantly longer mOS [IMbrave-IN 15.0 months (95% CI: 10.7 – 19.3) *versus* IMbrave-OUT 6.0 months (95% CI: 3.2–8.9; *p* < 0.001)] and a significantly longer mPFS [IMbrave-IN 8.7 months (95% CI: 5.9–11.5) *versus* IMbrave-OUT 3.7 months (95% CI: 2.7–4.7); *p* < 0.001] (Figure 1(a) and (b)). Radiologic tumor response assessment was available for 119 patients (81.0%) (Figure 2(a)). A complete response (CR) was reported in 3 patients (2.0%) and 27 (18.4%) had a partial radiographic response (PR), resulting in an ORR of 20.4%. Stable disease (SD) was reported in 46 patients (31.3%), resulting in a disease control rate (DCR) of 51.7%. Progressive disease (PD) was reported in 43 patients (29.3%). There were no significant differences for ORR and DCR between the IMbrave-IN and IMbrave-OUT subgroups. Median OS was similar in patients with a radiographic CR/PR or SD [CR and PR 15.9 months (95% CI: 13.1–18.7) *versus* SD 13.4 months (95% CI: not estimable (NE); *p* = 0.53)]. In contrast, patients with PD as best radiologic response reached an mOS of only 6.4 months (95% CI: 5.3–7.5), which was significantly lower than in patients with CR/PR (*p* < 0.001) or SD (*p* < 0.001) (Figure 2(b)).

In univariable analysis, CPS, ALBI grade, previous locoregional or systemic anticancer treatments, baseline AFP level ⩾400 ng/ml, macrovascular invasion, extrahepatic spread, or etiology [viral *versus* alcoholic steatohepatitis (ASH) *versus* non-alcoholic steatohepatitis (NASH)] were not predictive for ORR or DCR (data not shown).

**Figure 3.**
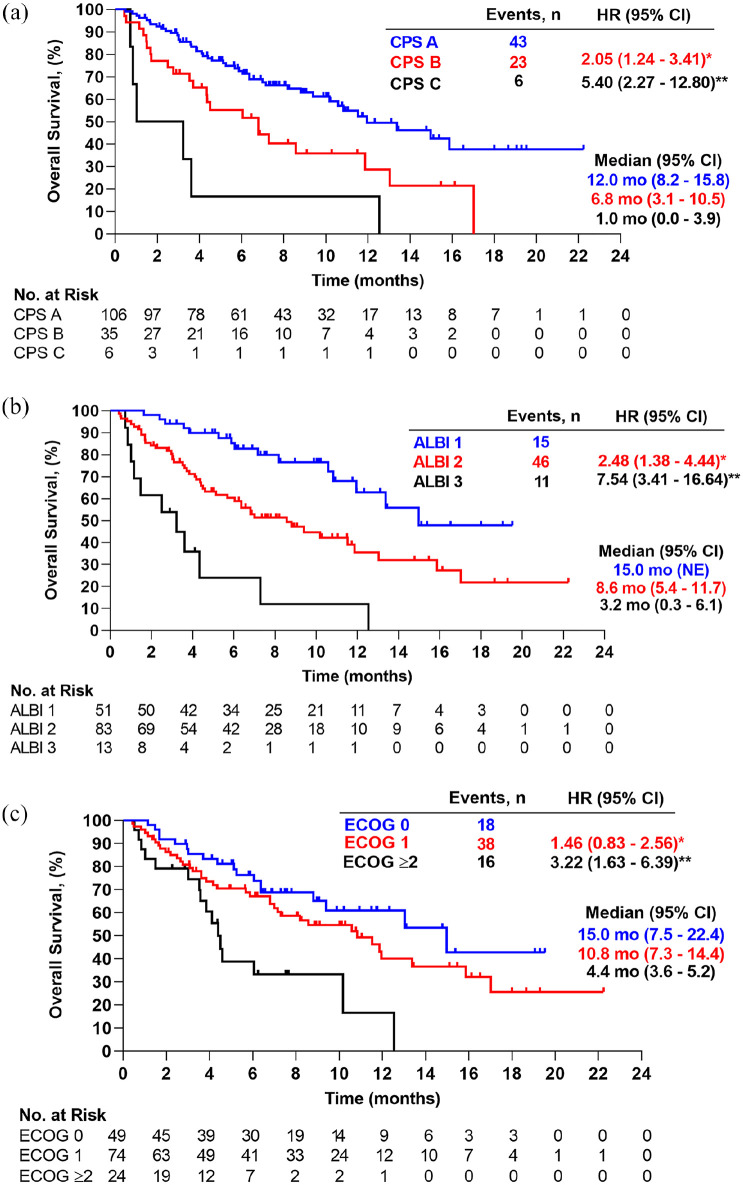
Kaplan–Meier analysis of overall survival by (a) Child–Pugh score, (b) ALBI grade, and (c) ECOG score in patients treated with atezolizumab/bevacizumab. ALBI, albumin-bilirubin; CI, confidence interval; CPS, Child–Pugh score; ECOG, Eastern Cooperative Oncology Group; HR, hazard ratio; mo, months; NE, not estimable; No., number. (a) *comparing HR of death between CPS B and CPS A; **comparing HR of death between CPS C and CPS A. (b) *comparing HR of death between ALBI 2 and ALBI 1; **comparing HR of death between ALBI 3 and ALBI 1. (c) *comparing HR of death between ECOG 1 and ECOG 0; **comparing HR of death between ECOG ⩾2 and ECOG 0.

To determine the impact of liver function on OS, Kaplan–Meier curves were stratified according to CPS or ALBI grade at baseline. Both scoring systems revealed significant differences for mOS: CPS A patients reached an mOS of 12.0 months (95% CI: 8.2–15.8) compared to 6.8 months (95% CI: 3.1–10.5; *p* = 0.04) in the CPS B group. Median OS was 1.0 month (95% CI: 0.0–3.9; *p* < 0.001) for the few CPS C patients who were included in the study (*n* = 6) (Figure 3(a)). According to ALBI grade, which achieves a more granular discrimination especially within the CPS A group (Table 2), ALBI grade 1 patients reached an mOS of 15.0 months (95% CI: NE), whereas mOS in ALBI grade 2 patients was significantly lower with 8.6 months (95% CI: 5.4 – 11.7; *p* = 0.002), followed by 3.2 months (95% CI: 0.3–6.1; *p* < 0.001) in the ALBI grade 3 group (Figure 3(b)). Median OS was also significantly longer for patients with preserved ECOG performance status [ECOG 0 with 15.0 months (95% CI: 7.5 – 22.4) *versus* ECOG ⩾2 with 4.4 months (95% CI: 3.6–5.2; *p* < 0.001)]; there was no significant difference in mOS between ECOG 0 and ECOG 1 patients (10.8 months for ECOG 1 (95% CI: 7.3 – 14.4; *p* = 0.188)] (Figure 3(c)). Prior systemic treatment and underlying liver disease (viral *versus* ASH *versus* NASH) did not affect OS or PFS in our cohort (Supplemental Figures S1 and S2).

**Figure 4.**
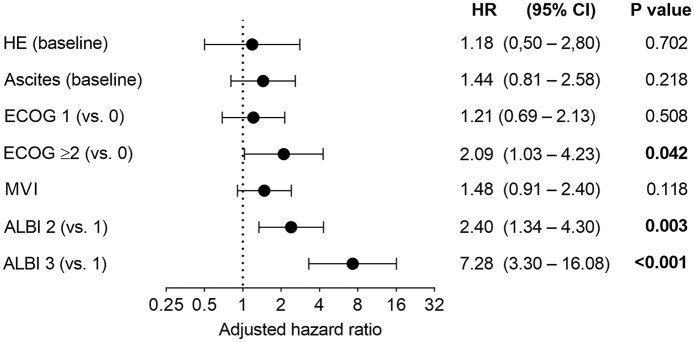
Multivariable analysis of overall survival with adjusted hazard ratios. ALBI, albumin-bilirubin; CI, confidence interval; ECOG, Eastern Cooperative Oncology Group; HR, hazard ratio (adjusted); HE, hepatic encephalopathy; MVI, macrovascular invasion.

A more extensive univariable Kaplan–Meier survival analysis and HRs associated with overall survival benefit in univariable Cox regression are depicted in Supplemental Table S2. In multivariable analysis, baseline ALBI grade and ECOG score were identified as independent prognostic predictors [ALBI grade 2 *versus* 1; HR: 2.40 (95% CI: 1.34–4.30; *p* = 0.003), ALBI grade 3 *versus* 1; HR: 7.28 (95% CI: 3.30–16.08; *p* < 0.001), and ECOG ⩾2 *versus* 0; HR: 2.09 (95% CI: 1.03–4.23; *p* = 0.042)], respectively (Figure 4).

### Safety

Table 4 summarizes the frequency of CTCAE grade ⩾3 AEs regardless of cause reported in all 147 patients after treatment initiation with atezolizumab/bevacizumab. Seventeen patients (11.6%) died during treatment due to gastrointestinal (GI) bleeding (variceal upper GI bleeding *n* = 5; non-variceal GI bleeding *n* = 1), immune-related adverse events (immune-mediated hepatitis *n* = 2), or hepatic failure (*n* = 9). No significant differences were seen in the frequency of non-liver-related toxicity between IMbrave-IN and IMbrave-OUT patients. Six non-fatal bleeding events [peptic ulcer bleeding (*n* = 2), tumor bleeding (*n* = 3) and intracranial bleeding event (*n* = 1)] led to the termination of bevacizumab. Severe immune-related AEs (IR-AE) were reported in 15 patients (10.2%), which led to treatment discontinuation in 6 patients (4.1%). All patients with severe IR-AEs received subsequent therapy with systemic steroids. Two patients with immune-mediated hepatitis in the IMbrave-OUT subgroup developed severe ascites and died 6 weeks after starting steroids. There were no significant differences in frequency of IR-AEs between the IMbrave-IN and IMbrave-OUT subgroups.

**Table 4.** Frequency of adverse events of CTCAE grade ⩾3, immune-related adverse events, and worsening of liver function according to patients’ eligibility for the IMbrave150 trial.

Frequency of CTCAE grade ⩾3	IMbrave-IN (*n* = 74) *n* (%)	IMbrave-OUT (*n* = 73) *n* (%)	Total (*n* = 147) *n* (%)
Bleeding complication	11 (14.9)	10 (13.7)	21 (14.2)
Variceal upper GI bleeding	3 (4.1)	3 (4.1)	6 (4.1)
Non-variceal GI bleeding	3 (4.1)	2 (2.7)	5 (3.4)
Non-GI bleeding	5 (6.8)	5 (6.8)	10 (6.8)
Thromboembolism	4 (5.4)	4 (5.4)	8 (5.4)
Pulmonary embolism	3 (4.1)	2 (2.7)	5 (3.4)
Myocardial infarction	0 (0)	1 (1.4)	1 (0.7)
Portal vein thrombosis	1 (1.4)	1 (1.4)	2 (1.4)
Immune-related AEs	10 (13.5)	5 (6.8)	15 (10.2)
Hepatitis	2 (2.7)	2 (2.7)	4 (2.7)
Rheumatic and musculoskeletal	4 (5.4)	1 (1.4)	5 (3.4)
Hypophysitis	0 (0.0)	1 (1.4)	1 (0.7)
Colitis	1 (1.4)	0 (0.0)	1 (0.7)
Cholangitis	1 (1.4)	1 (1.4)	2 (1.4)
Nephritis	1 (1.4)	0 (0.0)	1 (0.7)
Mucositis	1 (1.4)	0 (0.0)	1 (0.7)
Seizures	0 (0.0)	1 (1.4)	1 (0.7)
Worsening of liver function	8 (10.8)	39 (53.4)	47 (32.0)
New onset/aggravation of ascites (any grade):	7 (9.5)	29 (39.7)	36 (24.5)
Large-volume ascites (grade ⩾3)	2 (2.7)	14 (19.2)	16 (10.9)
New onset/aggravation of HE (any grade):	1 (1.4)	10 (13.7)	11 (7.5)
High-grade HE (grade ⩾3)	0 (0)	6 (8.2)	6 (4.1)

AE, adverse event; CTCAE, Common Terminology Criteria for Adverse Events; GI, gastrointestinal; HE, hepatic encephalopathy.

Indicative of a higher risk of liver decompensation, patients in the IMbrave-OUT group were significantly more likely to develop new-onset ascites or deterioration of preexisting ascites compared with patients in the IMbrave-IN group (39.7% *versus* 9.5%, *p* < 0.001). Ascitic decompensations were also more severe in the IMbrave-OUT patients (19.2% *versus* 2.7%, *p* < 0.001). In addition, *de novo* hepatic encephalopathy (HE) and severe episodes of HE were more common in IMbrave-OUT patients (13.7% *versus* 1.4%, *p* = 0.004, and 8.2% *versus* 0.0%, *p* = 0.004, respectively). Twelve patients (8.2%) developed severe hepatic failure requiring hospitalization, and outcome was fatal in 9 of them (6.1%). One patient received a transvenous intrahepatic portosystemic shunt (TIPSS) and was able to resume treatment afterward, whereas atezolizumab/bevacizumab was permanently discontinued in the remaining two patients. Overall, patients with ALBI grade ⩾2 and patients with ECOG ⩾2 were more prone to develop ascites [9.8% in ALBI grade 1 *versus* 32.6% in ALBI grade ⩾ 2 (*p* = 0.002); 19.5% in patients with ECOG 0 and 1 *versus* 52.2% in ECOG ⩾2 (*p* < 0.001)] (Supplemental Figure S3A). Patients with impaired liver function and impaired performance status were also at higher risk of developing HE [0.0% in ALBI grade 1 *versus* 11.5% in ALBI grade ⩾2 (*p* = 0.009); 3.3% in patients with ECOG 0 and 1 *versus* 29.2% in ECOG ⩾2 (*p* < 0.001)] (Supplemental Figure S3B). Etiology (viral *versus* nonviral), presence of macrovascular invasion, varices at baseline, and prior local or systemic treatment were not associated with increased risk of developing ascites or HE (data not shown).

To evaluate the impact of combination therapy with atezolizumab/bevacizumab on liver function, changes in ALBI score between baseline and after 8–12 weeks of treatment were assessed in all patients with available data (*n* = 124, 84.4%) (Supplemental Figure S4). ALBI score significantly worsened from baseline mean score of −2.67 ± 0.46 to −2.47 ± 0.58 (*p* < 0.001) in IMbrave-IN patients, resulting in a shift from baseline ALBI grade 1 to grade 2 in 14 of 37 patients and from baseline ALBI grade 2 to grade 3 in 4 of 29 patients. Similarly, IMbrave-OUT patients experienced a significant worsening from mean baseline ALBI score of −1.91 ± 0.61 to −1.59 ± 0.74 after 8–12 weeks of treatment (*p* < 0.001), leading to a shift from ALBI grade 1 to grade 2 in 3 of 6 patients and from ALBI grade 2 to grade 3 in 15 of 42 patients. Overall, IMbrave-IN and IMbrave—OUT subgroups were equally prone to deterioration of liver function according to ALBI grade after 8–12 weeks of treatment with atezolizumab/bevacizumab (*p* = 0.645).

## Discussion

The recent approval of atezolizumab/bevacizumab has established a new standard of care for the systemic treatment of advanced HCC.^10^ In this study, we evaluated the benefit of the combination therapy in a real-word cohort that included patients from six tertiary care centers in Germany and Austria. To our knowledge, this is the first multicenter study that reports efficacy and safety outcomes in patients with advanced HCC receiving atezolizumab/bevacizumab with impaired liver function CPS ⩾B7 and prior systemic therapies.

Our study confirmed the activity of the ICI-based combination in patients who meet the inclusion criteria of the IMbrave150 study in a real-world setting. Efficacy analysis in this real-world cohort revealed an ORR of 25.7% and a DCR of 60.8% for IMbrave-IN patients with an mPFS of 8.7 months, in line with the reported outcomes in the updated report of the pivotal trial (ORR 29.8%, DCR 74.0%, and median PFS of 6.9 months).^27^ Median OS for IMbrave-IN patients was 15.0 months after a maximum follow-up period of 22.2 months. In the IMbrave-OUT group, radiologic response rate was 20.4%, but with an mPFS of only 3.7 months and an mOS of 6.0 months (*p* < 0.001 for OS and *p* < 0.001 for PFS). Thus, our findings confirm the benefit of the inclusion criteria of clinical trials to achieve the best outcomes with systemic therapies in patients with advanced HCC independent of the underlying liver disease.

Currently, atezolizumab/bevacizumab is only approved in the first-line setting based on the pivotal phase III trial, and its efficacy in subsequent lines is less well defined. In agreement with a previous report, our data suggest that the efficacy of atezolizumab/bevacizumab might be independent of the treatment line.^28^

An impaired liver function often poses a severe challenge to the management of patients with HCC. Therefore, we were interested in the efficacy of atezolizumab/bevacizumab in patients with impaired liver function.^29^ Baseline ALBI grade has been confirmed as a prognostic indicator in HCC phase III studies with sorafenib^30,31^ and lenvatinib^32^ in the first-line setting as well as with cabozantinib,^33^ regorafenib^34^ and ramucirumab^35^ in the second-line setting. A similar impact was not only observed for pembrolizumab in the KEYNOTE-240 study,^36^ but also more recently in the IMbrave150 study.^37^ Of note, mOS in the ALBI grade 1 cohort of the phase III study was not reached, compared with 14.4 months in patients with ALBI grade 2. The significant impact on mOS in patients treated with this ICI-based combination was even more evident in our real-world cohort, in which mOS of patients with a CPS ⩾B7 and an ALBI grade ⩾2 was only 4.5 months and 6.8 months, respectively. These data are in line with previous real-world studies with ICIs, in which the mOS ranged only up to 9.6 months in patients with CPS ⩾B7 or ALBI grade ⩾2.^21–24^ The broad range in mOS reported from real-world cohorts likely reflects the heterogeneity of the patient populations in respect to baseline characteristics such as performance status, macrovascular invasion, presence of ascites, and baseline liver function.^29,38^ Our multivariable analysis confirmed ALBI grade and ECOG performance status as independent prognostic predictors of survival.^21,25^ Therefore, our findings support the concept that stratification according to liver function such as ALBI score is of immediate prognostic value.

In respect to biomarkers for decision-making in advanced HCC, a recent study suggested that clinicians might need to consider the underlying liver disease for treatment selection.^39^ The authors provided preclinical evidence that NASH-related HCCs might benefit less from immune checkpoint inhibition, compared with viral-induced HCCs. In our real-world cohort, the underlying liver disease (viral *versus* ASH *versus* NASH) did not have a significant effect on mPFS and mOS.

Regarding safety outcomes, the incidence of grade ⩾3 AEs in the present study was 45.6%, which was lower than reported for the experimental arm of the IMbrave150 trial (56.5%).^10^ There was no significant difference in the incidence of non-hepatic grade ⩾3 AEs between the IMbrave-IN and IMbrave-OUT group, but patients in the IMbrave-OUT were at higher risk of treatment discontinuation related to deterioration of liver function. Due to the retrospective nature of this study and the possible underreporting of CTCAEs, the safety analysis was focused on severe events and/or those that led to treatment discontinuation or hospitalization. Our data revealed that only about three out of four patients had received an upper GI endoscopy to detect esophageal varices prior to initiation of atezolizumab/bevacizumab. Six bleeding events from esophageal varices were recorded in our real-world cohort, and we therefore strongly support the recommendation that all patients should undergo an upper GI endoscopy before treatment initiation with bevacizumab.^40^ While the incidence of severe bleeding (including fatal events) was similar to the data reported for the combination arm of the IMbrave150 trial,^10^ the rate of immune-related hepatitis was slightly higher in our study (2.7%) compared with the phase III trial (0.6%). Other studies have reported onset of immune-mediated hepatitis in 1–4% of patients with advanced HCC under monotherapy with PD-1 inhibitors.^41,42^ Of note, diagnosing immune-mediated hepatitis can be clinically challenging in patients with hepatic malignancies and underlying liver diseases.^41,43^

Another key aspect of this study was to evaluate changes in liver function and to assess the likelihood of liver decompensation under treatment with atezolizumab/bevacizumab. We observed a deterioration of liver function according to the ALBI score in both IMbrave-IN and IMbrave-OUT subgroups. New onset of ascites and/or HE in the IMbrave-IN group (9.5% and 1.4%) was similar to what was reported in the IMbrave150 trial (7.0% and 1.5%), whereas IMbrave-OUT patients were more likely to experience decompensation of liver function with the occurrence of large-volume ascites (19.2%) and/or high-grade HE (8.2%) requiring hospitalization. Patients with ALBI grade ⩾2 (*p* = 0.002) and decreased performance status ECOG ⩾2 (*p* < 0.001) at baseline were at highest risk for ascitic decompensation and development of HE. Of note, ascitic decompensation and HE are generally considered to be less common in patients receiving mono-immunotherapy^20–22,44^ than in patients under treatment with tyrosine kinase inhibitors.^16,45–47^ However, AEs linked to portal hypertension were more frequently reported for atezolizumab/bevacizumab, suggesting a potential association with the anti-angiogenic treatment.^48^ Overall, our data strongly suggest that patients with impaired liver function and reduced ECOG performance status face an increased risk of liver-related complications such as ascites and HE. These patients require close monitoring for early detection of clinically relevant deterioration.

Our study has several limitations, such as its retrospective nature and an inherent sampling bias due to the limited size of the cohort. Of note, the high proportion of patients with ASH in our cohort exceeds that of other studies (and especially studies with recruitment in Asia),49 where viral hepatitis is the predominant cause of HCC. In addition, response data based on radiologic imaging were evaluated by the local radiologist without central review. Also, subsequent therapies after atezolizumab/bevacizumab were not documented in our cohort, and therefore the impact of sequential therapies on mOS remains enigmatic.

In summary, our study confirms the anti-tumor activity of atezolizumab/bevacizumab in a real-world cohort with encouraging survival outcomes and acceptable toxicity, as previously reported in the pivotal IMbrave150 study. Clinical efficacy was observed independent of prior systemic therapy, thus indicating that atezolizumab/bevacizumab maintains a meaningful activity also in second-line therapy. However, we add a note of caution to the use of atezolizumab/bevacizumab in patients with symptoms of liver decompensation and recommend close monitoring of patients with significantly impaired liver function. Prospective clinical trials that include patients with compromised performance status and/or liver function are needed to validate our findings and to optimize treatment strategies in this clinically relevant patient subgroup.

## Supplemental Material

sj-docx-1-tam-10.1177_17588359221080298 – Supplemental material for Atezolizumab and bevacizumab in patients with advanced hepatocellular carcinoma with impaired liver function and prior systemic therapy: a real-world experience Click here for additional data file. Supplemental material, sj-docx-1-tam-10.1177_17588359221080298 for Atezolizumab and bevacizumab in patients with advanced hepatocellular carcinoma with impaired liver function and prior systemic therapy: a real-world experience by Tiago de Castro, Leonie S. Jochheim, Melanie Bathon, Sabrina Welland, Bernhard Scheiner, Kateryna Shmanko, Daniel Roessler, Najib Ben Khaled, Matthias Jeschke, Johannes M. Ludwig, Jens U. Marquardt, Arndt Weinmann, Matthias Pinter, Christian M. Lange, Arndt Vogel and Anna Saborowski in Therapeutic Advances in Medical Oncology

sj-docx-2-tam-10.1177_17588359221080298 – Supplemental material for Atezolizumab and bevacizumab in patients with advanced hepatocellular carcinoma with impaired liver function and prior systemic therapy: a real-world experience Click here for additional data file. Supplemental material, sj-docx-2-tam-10.1177_17588359221080298 for Atezolizumab and bevacizumab in patients with advanced hepatocellular carcinoma with impaired liver function and prior systemic therapy: a real-world experience by Tiago de Castro, Leonie S. Jochheim, Melanie Bathon, Sabrina Welland, Bernhard Scheiner, Kateryna Shmanko, Daniel Roessler, Najib Ben Khaled, Matthias Jeschke, Johannes M. Ludwig, Jens U. Marquardt, Arndt Weinmann, Matthias Pinter, Christian M. Lange, Arndt Vogel and Anna Saborowski in Therapeutic Advances in Medical Oncology

sj-tif-1-tam-10.1177_17588359221080298 – Supplemental material for Atezolizumab and bevacizumab in patients with advanced hepatocellular carcinoma with impaired liver function and prior systemic therapy: a real-world experience Click here for additional data file. Supplemental material, sj-tif-1-tam-10.1177_17588359221080298 for Atezolizumab and bevacizumab in patients with advanced hepatocellular carcinoma with impaired liver function and prior systemic therapy: a real-world experience by Tiago de Castro, Leonie S. Jochheim, Melanie Bathon, Sabrina Welland, Bernhard Scheiner, Kateryna Shmanko, Daniel Roessler, Najib Ben Khaled, Matthias Jeschke, Johannes M. Ludwig, Jens U. Marquardt, Arndt Weinmann, Matthias Pinter, Christian M. Lange, Arndt Vogel and Anna Saborowski in Therapeutic Advances in Medical Oncology

sj-tif-2-tam-10.1177_17588359221080298 – Supplemental material for Atezolizumab and bevacizumab in patients with advanced hepatocellular carcinoma with impaired liver function and prior systemic therapy: a real-world experience Click here for additional data file. Supplemental material, sj-tif-2-tam-10.1177_17588359221080298 for Atezolizumab and bevacizumab in patients with advanced hepatocellular carcinoma with impaired liver function and prior systemic therapy: a real-world experience by Tiago de Castro, Leonie S. Jochheim, Melanie Bathon, Sabrina Welland, Bernhard Scheiner, Kateryna Shmanko, Daniel Roessler, Najib Ben Khaled, Matthias Jeschke, Johannes M. Ludwig, Jens U. Marquardt, Arndt Weinmann, Matthias Pinter, Christian M. Lange, Arndt Vogel and Anna Saborowski in Therapeutic Advances in Medical Oncology

sj-tif-3-tam-10.1177_17588359221080298 – Supplemental material for Atezolizumab and bevacizumab in patients with advanced hepatocellular carcinoma with impaired liver function and prior systemic therapy: a real-world experience Click here for additional data file. Supplemental material, sj-tif-3-tam-10.1177_17588359221080298 for Atezolizumab and bevacizumab in patients with advanced hepatocellular carcinoma with impaired liver function and prior systemic therapy: a real-world experience by Tiago de Castro, Leonie S. Jochheim, Melanie Bathon, Sabrina Welland, Bernhard Scheiner, Kateryna Shmanko, Daniel Roessler, Najib Ben Khaled, Matthias Jeschke, Johannes M. Ludwig, Jens U. Marquardt, Arndt Weinmann, Matthias Pinter, Christian M. Lange, Arndt Vogel and Anna Saborowski in Therapeutic Advances in Medical Oncology

sj-tif-4-tam-10.1177_17588359221080298 – Supplemental material for Atezolizumab and bevacizumab in patients with advanced hepatocellular carcinoma with impaired liver function and prior systemic therapy: a real-world experience Click here for additional data file. Supplemental material, sj-tif-4-tam-10.1177_17588359221080298 for Atezolizumab and bevacizumab in patients with advanced hepatocellular carcinoma with impaired liver function and prior systemic therapy: a real-world experience by Tiago de Castro, Leonie S. Jochheim, Melanie Bathon, Sabrina Welland, Bernhard Scheiner, Kateryna Shmanko, Daniel Roessler, Najib Ben Khaled, Matthias Jeschke, Johannes M. Ludwig, Jens U. Marquardt, Arndt Weinmann, Matthias Pinter, Christian M. Lange, Arndt Vogel and Anna Saborowski in Therapeutic Advances in Medical Oncology

sj-tif-5-tam-10.1177_17588359221080298 – Supplemental material for Atezolizumab and bevacizumab in patients with advanced hepatocellular carcinoma with impaired liver function and prior systemic therapy: a real-world experience Click here for additional data file. Supplemental material, sj-tif-5-tam-10.1177_17588359221080298 for Atezolizumab and bevacizumab in patients with advanced hepatocellular carcinoma with impaired liver function and prior systemic therapy: a real-world experience by Tiago de Castro, Leonie S. Jochheim, Melanie Bathon, Sabrina Welland, Bernhard Scheiner, Kateryna Shmanko, Daniel Roessler, Najib Ben Khaled, Matthias Jeschke, Johannes M. Ludwig, Jens U. Marquardt, Arndt Weinmann, Matthias Pinter, Christian M. Lange, Arndt Vogel and Anna Saborowski in Therapeutic Advances in Medical Oncology

sj-tif-6-tam-10.1177_17588359221080298 – Supplemental material for Atezolizumab and bevacizumab in patients with advanced hepatocellular carcinoma with impaired liver function and prior systemic therapy: a real-world experience Click here for additional data file. Supplemental material, sj-tif-6-tam-10.1177_17588359221080298 for Atezolizumab and bevacizumab in patients with advanced hepatocellular carcinoma with impaired liver function and prior systemic therapy: a real-world experience by Tiago de Castro, Leonie S. Jochheim, Melanie Bathon, Sabrina Welland, Bernhard Scheiner, Kateryna Shmanko, Daniel Roessler, Najib Ben Khaled, Matthias Jeschke, Johannes M. Ludwig, Jens U. Marquardt, Arndt Weinmann, Matthias Pinter, Christian M. Lange, Arndt Vogel and Anna Saborowski in Therapeutic Advances in Medical Oncology
