# Supplementary material for: Atezolizumab and bevacizumab in patients with advanced hepatocellular carcinoma with impaired liver function and prior systemic therapy: a real-world experience
Source: Ther Adv Med Oncol. 2022 Feb 26;14:17588359221080298. doi: 10.1177/17588359221080298 (PMC8891886; doi:10.1177/17588359221080298)
Supplement: sj-docx-1-tam-10.1177_17588359221080298 – Supplemental material for Atezolizumab and bevacizumab in patients with advanced hepatocellular carcinoma with impaired liver function and prior systemic therapy: a real-world experience [file sj-docx-1-tam-10.1177_17588359221080298.docx]

**Supplemental table S1.** Patient distribution according to medical center.

| Medical center | Patients  no., (%) |
| --- | --- |
| Hannover Medical School, Hannover, Germany | 58 (37.4) |
| Essen University Hospital, Essen, Germany | 45 (29.0) |
| University Medical Center of the Johannes Gutenberg University Mainz, Mainz, Germany | 22 (14.2) |
| Medical University of Vienna, Austria | 15 (9.7) |
| University Hospital of the Ludwig-Maximilians-University Munich, Munich, Germany | 11 (7.1) |
| University Hospital Schleswig-Holstein, Campus Lübeck, Lübeck, Germany | 4 (2.6) |
| Total | 155 (100) |

No., number.
