# Supplementary material for: Atezolizumab and bevacizumab in patients with advanced hepatocellular carcinoma with impaired liver function and prior systemic therapy: a real-world experience
Source: Ther Adv Med Oncol. 2022 Feb 26;14:17588359221080298. doi: 10.1177/17588359221080298 (PMC8891886; doi:10.1177/17588359221080298)
Supplement: sj-docx-2-tam-10.1177_17588359221080298 – Supplemental material for Atezolizumab and bevacizumab in patients with advanced hepatocellular carcinoma with impaired liver function and prior systemic therapy: a real-world experience [file sj-docx-2-tam-10.1177_17588359221080298.docx]

**Supplemental table S2.** Univariable analyses of overall survival using log-rank test and Cox regression with hazard ratios of death in patients treated with atezolizumab/bevacizumab**.**

|  | no. of events/ no. at risk | mOS  months (95% CI) | HR (95% CI) | P-Value |
| --- | --- | --- | --- | --- |
| BCLC^+^ |  |  |  |  |
| Stage B | 8/23 | 11.9 (NE) |  |  |
| Stage C | 57/116 | 10.8 (7.4 – 14.3) | 1.55 (0.74 – 3.24)* | .250 |
| Stage D | 6/7 | 3.2 (0.0 – 8.9) | 5.27 (1.82 – 15.31)** | **.002** |
| Etiology |  |  |  |  |
| Viral | 25/50 | 8.6 (3.1 – 14.0) |  |  |
| Nonviral | 47/97 | 11.5 (9.5 – 13.5) | 0.90 (0.56 – 1.47) | .683 |
| EHS |  |  |  |  |
| No EHS | 40/81 | 10.8 (8.0 – 13.7) |  |  |
| EHS | 32/65 | 10.2 (3.8 – 16.5) | 1.01 (0.65 – 1.64) | .898 |
| MVI |  |  |  |  |
| No MVI | 43/97 | 11.9 (9.4 – 14.3) |  |  |
| MVI | 29/48 | 6.8 (1.3 – 12.3) | 1.75 (1.09 – 2.82) | **.022** |
| Ascites (baseline) |  |  |  |  |
| No ascites | 46/112 | 13.0 (10.1 – 16.0) |  |  |
| Ascites | 26/35 | 4.4 (3.2 – 5.6) | 2.46 (1.52 – 3.99) | **<.001** |
| HE (baseline) |  |  |  |  |
| No HE | 60/134 | 11.9 (9.3 – 14.4) |  |  |
| HE | 12/13 | 4.3 (2.8 – 5.8) | 2.59 (1.39 – 4.81) | **.003** |
| AFP (baseline) |  |  |  |  |
| <400 ng/ml | 41/93 | 11.9 (9.1 – 14.6) |  |  |
| ≥400 ng/ml | 30/52 | 8.2 (2.4 – 14.0) | 1.41 (0.88 – 2.27) | .150 |
| Prior local treatment |  |  |  |  |
| No prior local treatment | 41/81 | 10.2 (6.0 – 14.4) |  |  |
| Prior local treatment | 31/66 | 11.5 (7.6 – 15.4) | 0.86 (0.54 – 1.37) | .527 |

BCLC, Barcelona Classification Liver Cancer; MVI, macrovascular invasion; HE, hepatic encephalopathy; AFP, alpha-fetoprotein; no., number; mOS, median overall survival; NE, not estimable; HR, hazard ratio; CI, confidence interval; ^+^, BCLC stage A was omitted due to very low sample size (n=1); *, HR of death between BCLC stage C and Stage B; **, HR of death between BCLC stage D and stage B.
