# Supplementary figures and images for: Atezolizumab and bevacizumab in patients with advanced hepatocellular carcinoma with impaired liver function and prior systemic therapy: a real-world experience
Source: Ther Adv Med Oncol. 2022 Feb 26;14:17588359221080298. doi: 10.1177/17588359221080298 (PMC8891886; doi:10.1177/17588359221080298)

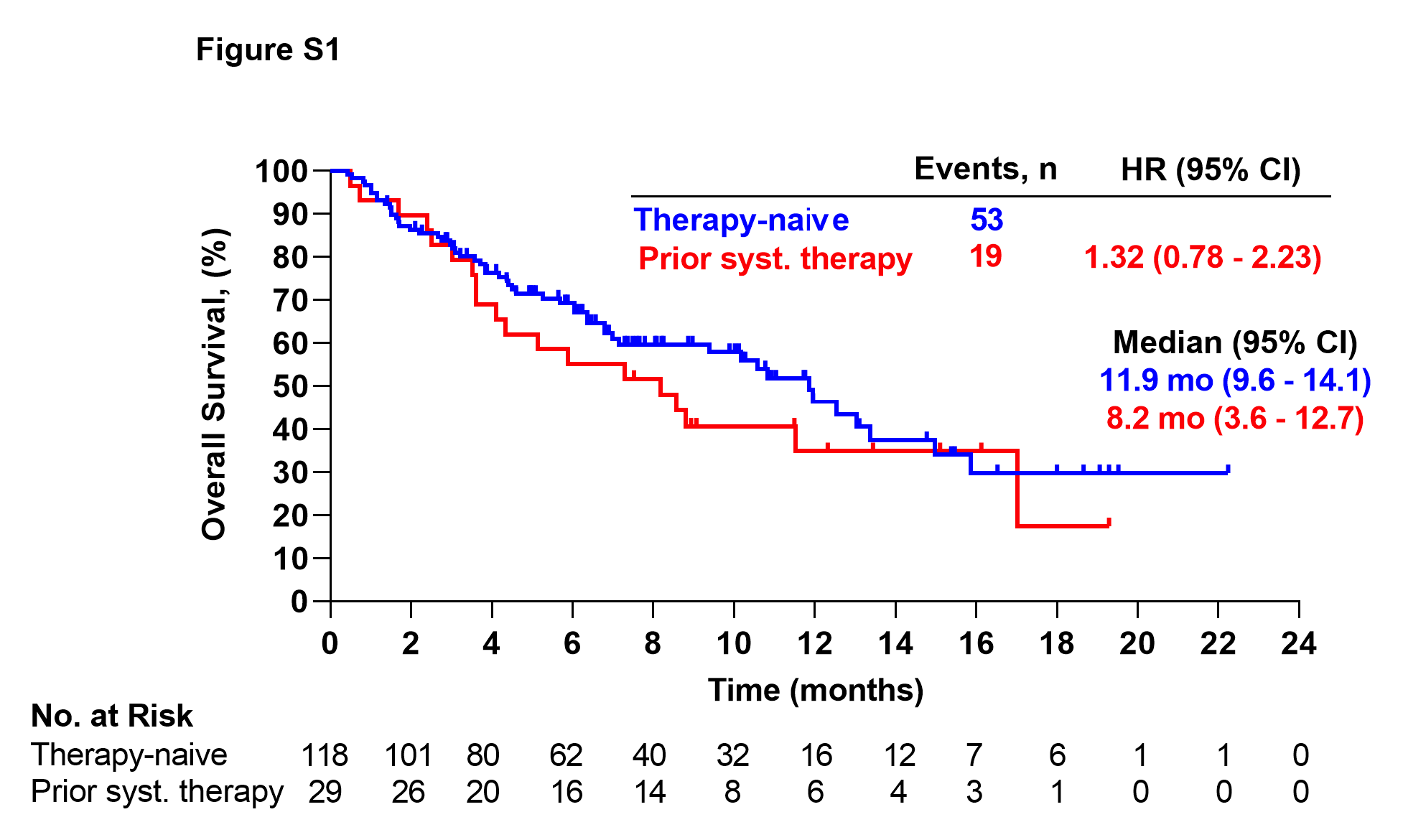

Supplement: sj-tif-1-tam-10.1177_17588359221080298 – Supplemental material for Atezolizumab and bevacizumab in patients with advanced hepatocellular carcinoma with impaired liver function and prior systemic therapy: a real-world experience [file sj-tif-1-tam-10.1177_17588359221080298.tif]

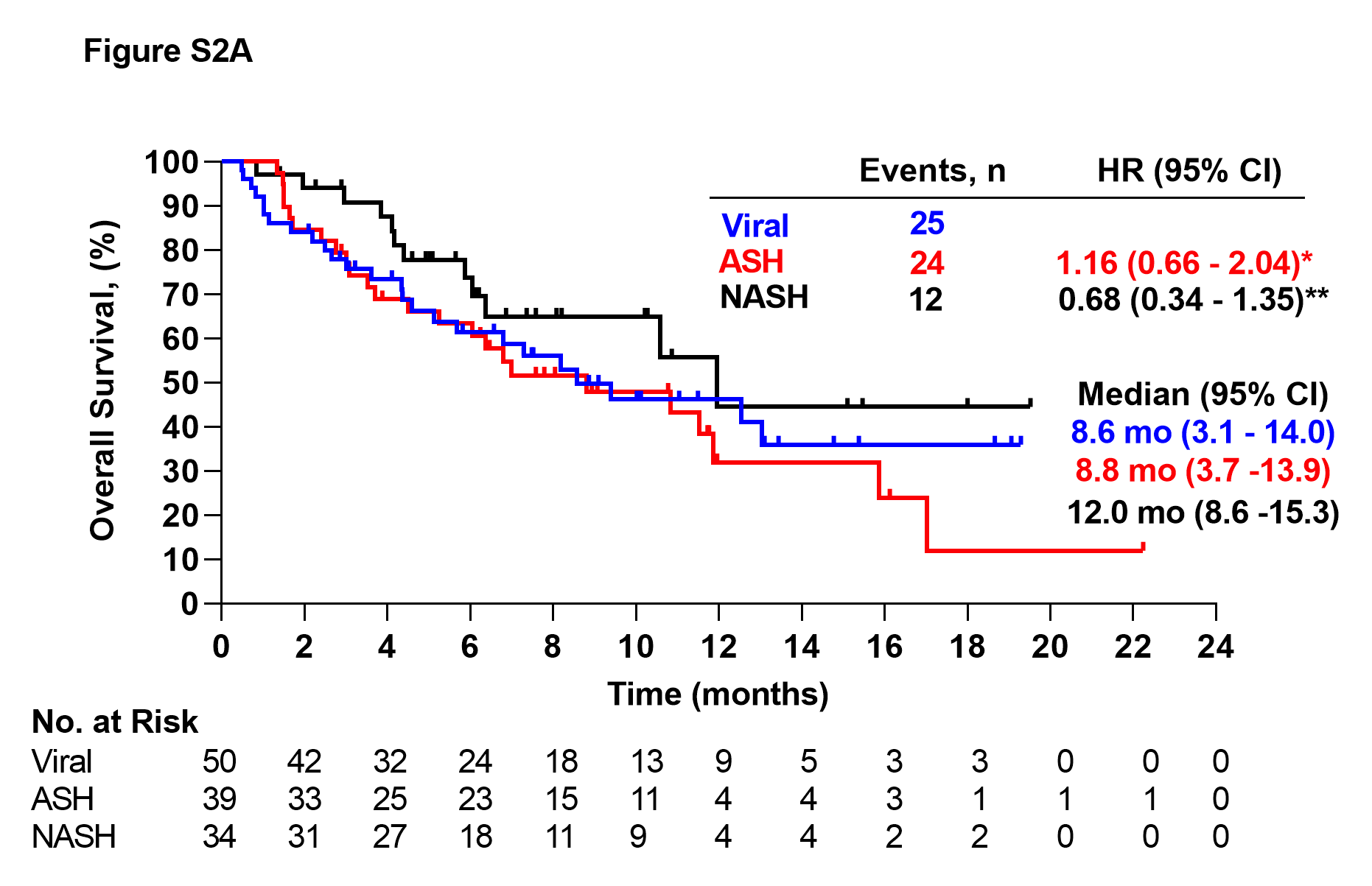

Supplement: sj-tif-2-tam-10.1177_17588359221080298 – Supplemental material for Atezolizumab and bevacizumab in patients with advanced hepatocellular carcinoma with impaired liver function and prior systemic therapy: a real-world experience [file sj-tif-2-tam-10.1177_17588359221080298.tif]

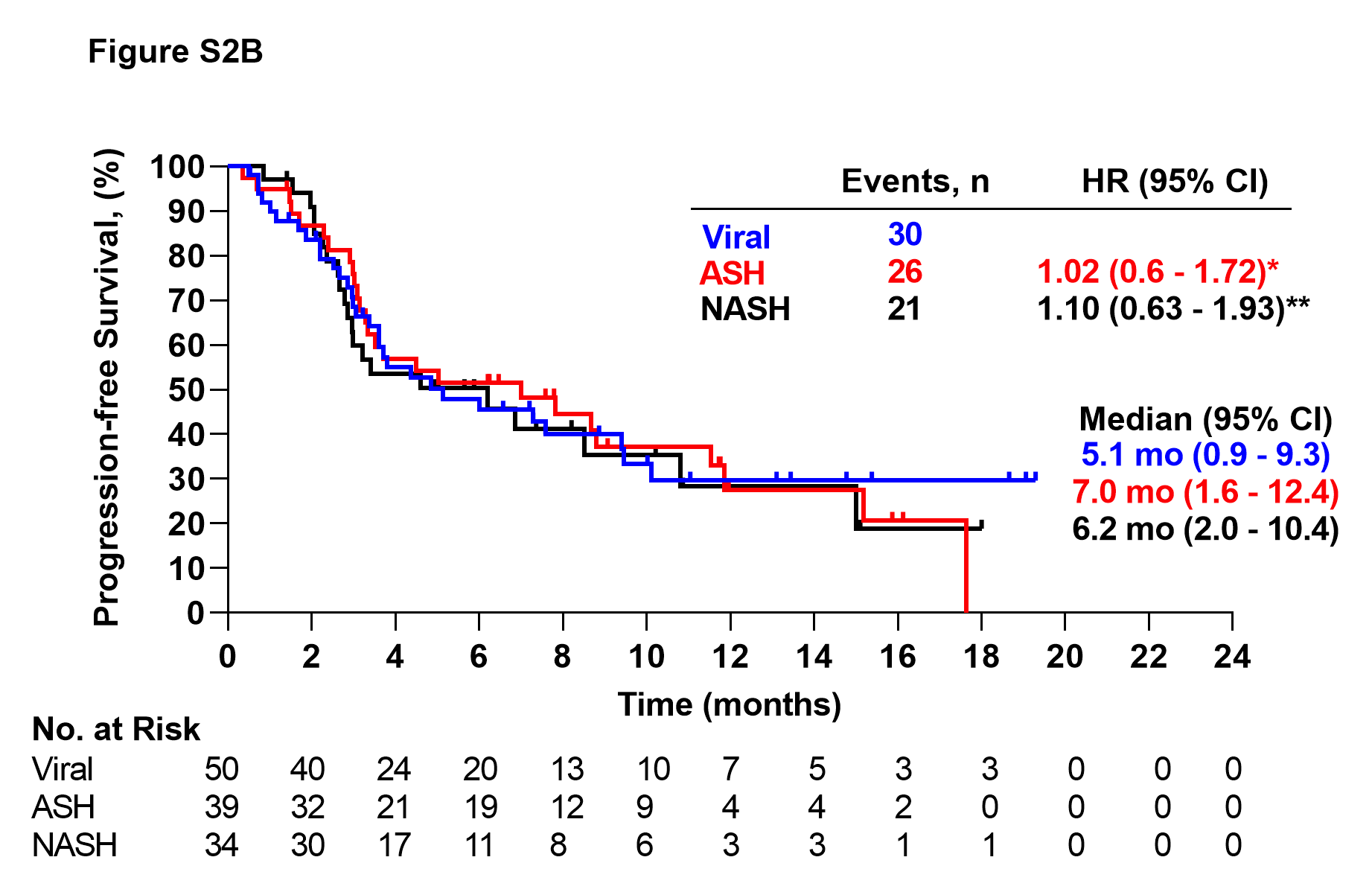

Supplement: sj-tif-3-tam-10.1177_17588359221080298 – Supplemental material for Atezolizumab and bevacizumab in patients with advanced hepatocellular carcinoma with impaired liver function and prior systemic therapy: a real-world experience [file sj-tif-3-tam-10.1177_17588359221080298.tif]

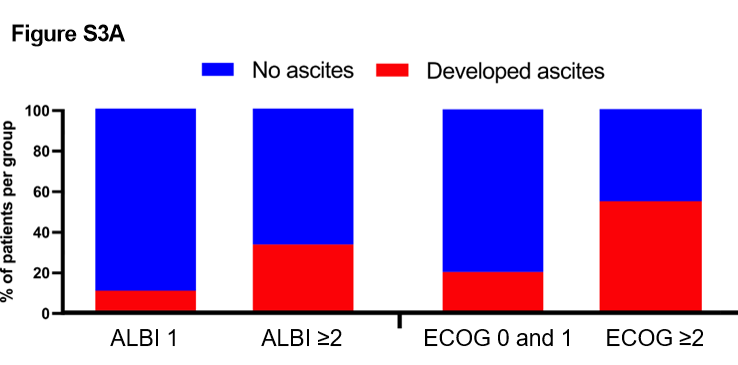

Supplement: sj-tif-4-tam-10.1177_17588359221080298 – Supplemental material for Atezolizumab and bevacizumab in patients with advanced hepatocellular carcinoma with impaired liver function and prior systemic therapy: a real-world experience [file sj-tif-4-tam-10.1177_17588359221080298.tif]

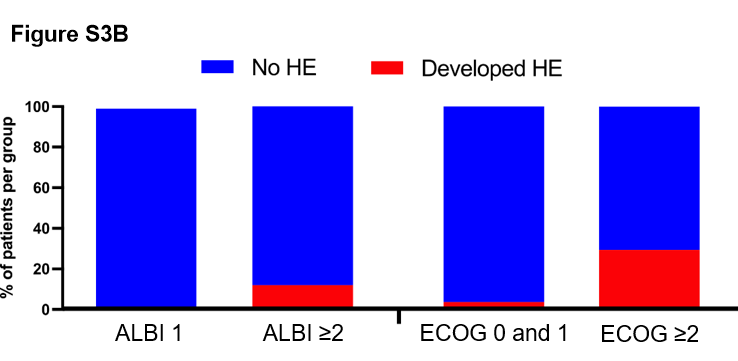

Supplement: sj-tif-5-tam-10.1177_17588359221080298 – Supplemental material for Atezolizumab and bevacizumab in patients with advanced hepatocellular carcinoma with impaired liver function and prior systemic therapy: a real-world experience [file sj-tif-5-tam-10.1177_17588359221080298.tif]

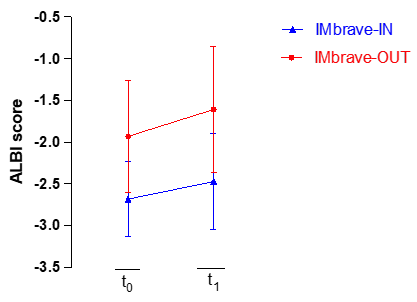

Supplement: sj-tif-6-tam-10.1177_17588359221080298 – Supplemental material for Atezolizumab and bevacizumab in patients with advanced hepatocellular carcinoma with impaired liver function and prior systemic therapy: a real-world experience [file sj-tif-6-tam-10.1177_17588359221080298.tif]
